# Electron Transport Disturbances and Neurodegeneration: From Albert Szent-Györgyi's Concept (Szeged) till Novel Approaches to Boost Mitochondrial Bioenergetics

**DOI:** 10.1155/2015/498401

**Published:** 2015-08-02

**Authors:** Levente Szalárdy, Dénes Zádori, Péter Klivényi, József Toldi, László Vécsei

**Affiliations:** ^1^Department of Neurology, Faculty of Medicine, Albert Szent-Györgyi Clinical Center, University of Szeged, Semmelweis u. 6, Szeged 6725, Hungary; ^2^Department of Physiology, Anatomy and Neuroscience, University of Szeged, Közép Fasor 52, Szeged 6726, Hungary; ^3^MTA-SZTE Neuroscience Research Group, Semmelweis u. 6, Szeged 6725, Hungary

## Abstract

Impaired function of certain mitochondrial respiratory complexes has long been linked to the pathogenesis of chronic neurodegenerative disorders such as Parkinson's and Huntington's diseases. Furthermore, genetic alterations of mitochondrial genome or nuclear genes encoding proteins playing essential roles in maintaining proper mitochondrial function can lead to the development of severe systemic diseases associated with neurodegeneration and vacuolar myelinopathy. At present, all of these diseases lack effective disease modifying therapy. Following a brief commemoration of Professor Albert Szent-Györgyi, a Nobel Prize laureate who pioneered in the field of cellular respiration, antioxidant processes, and the roles of free radicals in health and disease, the present paper overviews the current knowledge on the involvement of mitochondrial dysfunction in central nervous system diseases associated with neurodegeneration including Parkinson's and Huntington's disease as well as mitochondrial encephalopathies. The review puts special focus on the involvement and the potential therapeutic relevance of peroxisome proliferator-activated receptor-gamma coactivator 1-alpha (PGC-1*α*), a nuclear-encoded master regulator of mitochondrial biogenesis and antioxidant responses in these disorders, the transcriptional activation of which may hold novel therapeutic value as a more system-based approach aiming to restore mitochondrial functions in neurodegenerative processes.

## 1. The Man Amused by the Dance of Electrons

*“The fuel of life is electron, or more exactly, the energy it takes over from photons in photosynthesis, and gives up gradually while flowing through the cellular machinery.”*

This imagination originates from Professor Albert Szent-Györgyi, a Hungarian physician and biochemist, former chair of the Department of Medical Chemistry and the Department of Organic Chemistry at the University of Szeged from 1930 and 1935, respectively, until the end of World War II in 1945. His early research activities in Groningen and later in Cambridge conducted on biological combustion, cellular respiration, and energy production of plants lead to the discovery of a reducing substance called “hexuronic acid,” a substance that is able to lose and regain hydrogen atoms and capable of protecting plants from “browning,” an injury that he characterized as oxidative damage due to the excessive activity of an enzyme, peroxidase. This “antioxidant” substance was later proved by Szent-Györgyi to be equivalent with a potent antiscorbutic (antiscurvy) agent and was given the name ascorbic acid, currently widely known as vitamin C, which is most abundant in citrus fruits and paprika, an emblematic vegetable of Szeged. At this time, performing ongoing research in the field of biological respiration, Szent-Györgyi discovered and identified the catalysis of fumaric acid among other steps of the tricarboxylic acid cycle (also referred to as Szent-Györgyi–Krebs cycle, citric acid cycle), an essential component of cellular respiration that provides reducing equivalents for terminal oxidation and thereby energy production from metabolic products of dietary macromolecules. “*For his discoveries in connection with the biological combustion processes, with special reference to vitamin C and the catalysis of fumaric acid,*” Albert Szent-Györgyi was awarded Nobel Prize in Physiology or Medicine in 1937. In addition to his pioneering work in muscle research—including the discovery of actin and myosin proteins and the mechanism of their joint function—as well as the discovery of vitamin P (flavanone), his subsequent research interests focused on the interactions of proteins and free radicals and their role in regulating cell division and cancer development, and he published a number of books and papers about his findings and scientific theories of bioenergetics and bioelectronics and their roles in health and disease.

Following the imaginations of our honored predecessor, this paper reviews the concepts on the role of impairments in mitochondrial respiration and subsequent excessive oxidation in degenerative central nervous system (CNS) disorders, with special attention to recent findings related to alterations in transcriptional regulation of mitochondrial biogenesis and bioenergetics, and their potential therapeutic relevance.

## 2. Mitochondrial Respiration: The Proper Function of a Dangerous System

Mitochondria are membrane-bound intracellular organelles evolutionary originating from the endosymbiosis of an ancient aerobic alpha-proteobacterium with an early eukaryotic host cell [1]. Harboring their own maternally inherited, double-stranded, circular genome (mtDNA), supplemented by the presence of several ancillary, structural, and regulatory proteins encoded by the nuclear DNA (nDNA), mitochondria host a number of molecular processes essential for cellular life and death. These include processes related to the production of biologically utilizable energy, adaptive thermogenesis via the uncoupling of energy production, as well as the regulation of cellular calcium homeostasis, cell-cycle, and programmed cell death.

Energy production in the mitochondria is performed through the coupled function of pyruvate dehydrogenase complex (PDC), *β*-oxidation (processes essential in glycolytic and ketogenic metabolism, resp.), the Szent-Györgyi–Krebs cycle, and the terminal oxidation and oxidative phosphorylation (OXPHOS). While PDC, *β*-oxidation, and the Szent-Györgyi–Krebs cycle take place within the mitochondrial matrix, terminal oxidation and OXPHOS are linked to the function of respiratory complexes I–V (electron transport chain, ETC) embedded in the inner mitochondrial membrane. The process of energy production has been extensively reviewed elsewhere [2, 3]. Briefly, glucose is catabolized in the cytosol to yield pyruvate through multiple enzymatic steps of the glycolysis, which is then translocated into the mitochondria and metabolized to acetyl-coenzyme A (acetyl-CoA) by PDC. On the other hand, fatty acids are oxidized during the *β*-oxidation to acetyl-CoA entirely within the mitochondria (or in case of longer-chain fatty acids, initially within the peroxisomes). Mitochondrial acetyl-CoA subsequently enters the Szent-Györgyi–Krebs cycle to form reduced coenzyme NADH and succinate in multiple steps, which in turn provide electrons for respiratory complex I (NADH dehydrogenase) and complex II (succinate-ubiquinone oxidoreductase), respectively, using FMN and FAD as prosthetic groups, respectively. The electrons are then transported from both complexes via the mobile carrier coenzyme Q (ubiquinone) to complex III (ubiquinol-cytochrome *c* oxidoreductase) and flow through cytochrome *c* to reach complex IV (cytochrome *c* oxidase) to be oxidized by the final electron acceptor, oxygen. Notably, reducing equivalents produced during the glycolysis (in form of NADH) can also be translocated to the mitochondria via the malate/aspartate shuttle and the glycerol phosphate shuttle to provide NADH and FADH_2_, respectively, which in turn, similarly to NADH and FADH_2_ produced during *β*-oxidation, feed the ETC at complex I and coenzyme Q, respectively. Within the ETC, respiratory complexes are arranged in an electrochemical order, corresponding to their gradually increasing redox potential and electronegativity. The flow of electrons through the respiratory complexes provides energy used to pump out protons through complexes I, III, and IV (cytochrome *c* oxidase) to the intermembrane space. At the end of the downstream flow of electrons, molecular respiratory oxygen as the final electron acceptor is reduced by complex IV to form water molecule in a process known as terminal oxidation. The release of protons from the matrix develops a gradient of protons between the matrix and the intermembrane space also referred to as mitochondrial membrane potential (negative inside) as well as an electrochemical gradient (alkaline inside). Being impermeable to protons, the inner membrane works as a capacitator and an electric insulator. Therefore, the electrochemical drive to equalize the concentration of protons can be satisfied by the reentry of protons through respiratory complex V (H^+^-ATP synthase), the subsequent activation of which leads to the formation of ATP from ADP in a process called OXPHOS. The produced ATP represents a biologically available form of electrochemical energy, serving as the main energy-provider for eukaryotic cells (Mitchell's chemiosmotic hypothesis) [4]. The energy stored in mitochondrial membrane potential (also known as “proton motive force”) can also be utilized to generate heat or to import calcium or proteins into the mitochondrion via uncoupling the transport of electrons from ATP production, which processes serve adaptive purposes. The proportion of dietary calories burnt within the mitochondrion and allocated to energy production is referred to as “coupling efficiency.”

**Figure 1:**
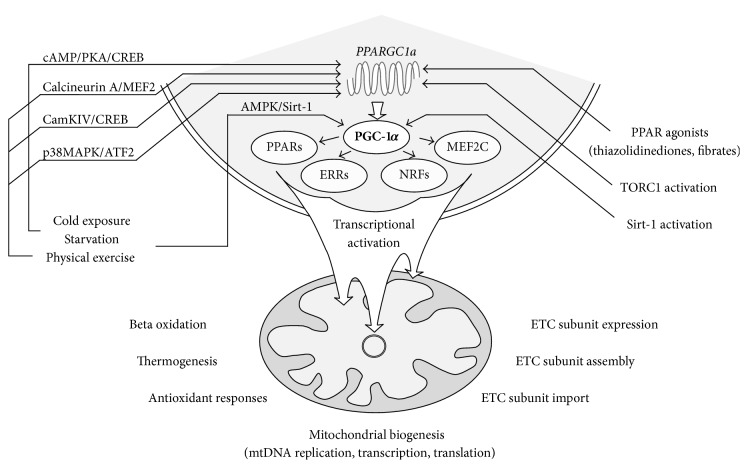
The PGC-1*α* cascade. Transcriptional upregulation or posttranslational activation of PGC-1*α* due to fasting, physical exercise, cold exposure, or pharmacological manipulations leads to the transcriptional activation of several nuclear-encoded proteins involved in mitochondrial functioning at multiple levels, including mitochondrial biogenesis, adaptive metabolism, antioxidant responses, and proper ETC assembly/import.

In multicellular organisms, the ability to adaptively regulate and activate mitochondrial biogenesis and functions in response to a variety of conditions is essential to maintain energetic homeostasis and cellular viability. Several lines of evidence obtained in the past decade suggest that peroxisome proliferator-activated receptor-gamma (PPAR*γ*) coactivator 1-alpha (PGC-1*α*), a nuclear-encoded coactivator of a wide range of transcriptional factors, plays a key role in the transcriptional cascade of such adaptive processes. PGC-1*α*-mediated coactivation of genes such as nuclear respiratory factor 1 and 2 (NRF-1, -2), PPARs, estrogen-related receptors (ERRs), and myocyte-specific enhancer factor 2C (MEF2C) leads to an increased expression of a wide range of proteins involved in mitochondrial transcription, replication, and the import and assembly of a number of nuclear-encoded respiratory complex subunits; furthermore, it boosts OXPHOS and thermoregulation in a tissue-dependent manner, enhances gluconeogenesis and fatty acid oxidation [5], and increase oxidative stress defense [6] (Figure 1). The inducing effect of* physical exercise* (mediated by calcineurin A-linked MEF2 activity, calcium/calmodulin-dependent protein kinase IV- (CaMKIV-) linked cyclic AMP (cAMP) response element-binding protein (CREB) activity, and a p38 mitogen-activated protein kinase- (MAPK-) linked activating transcription factor 2 (ATF-2) activity),* cold exposure* and* starvation* (mediated by catecholamine- and glucagon-induced cAMP elevation and a subsequent phosphorylation and activation of CREB by protein kinase A (PKA)) on PGC-1*α* expression is well documented [5]. Furthermore,* energy deprivation* through a high AMP/ATP ratio leads to an increased AMP-activated protein kinase (AMPK) activity and a subsequent phosphorylation of PGC-1*α* protein, priming PGC-1*α* for subsequent deacetylation and thereby activation by silent information regulator 2 homolog 1 (Sirt-1) [7, 8], the expression of which is also increased in conditions with energy shortage, such as, starvation or exercise, due to a high NAD^+^/NADH ratio [9]. These posttranslational modifications on PGC-1*α* play pivotal roles in adaptive mitochondrial biogenesis. The roles of impaired mitochondrial function and more recently a decreased function of the PGC-1*α* cascade in the pathogenesis of degenerative CNS disorders are of extensive research interest.

## 3. Mitochondrial Dysfunction, Reactive Oxygen, and Nitrogen Species

**Figure 2:**
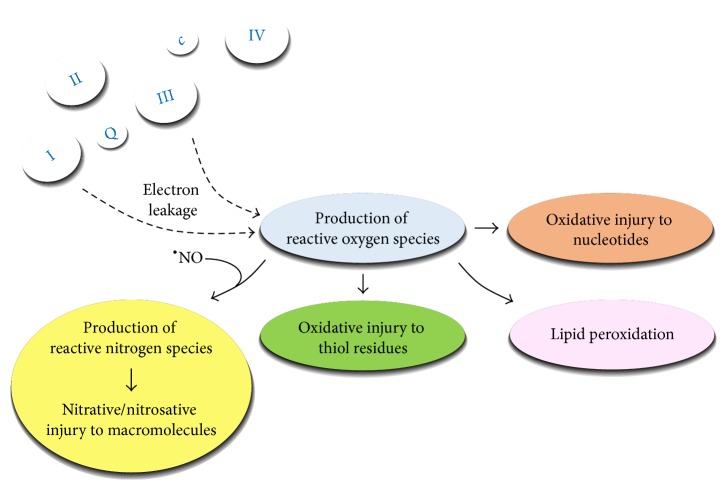
Schematic representation of the generation and the effects of free radicals within the mitochondria. The leakage of electrons from the mitochondrial ETC at complexes I and III results in the formation of superoxide anion. The high reactivity of this molecule evokes a harmful cascade mechanism including the formation of reactive oxygen and nitrogen species. The cascade mechanism deteriorates the functional groups of major components of all kinds of biomolecules (carbohydrates, lipids, proteins, and nucleic acids). In case of pronounced electron leakage or deficient antioxidant protection, a vicious circle of mitochondrial dysfunction develops.

**Figure 3:**
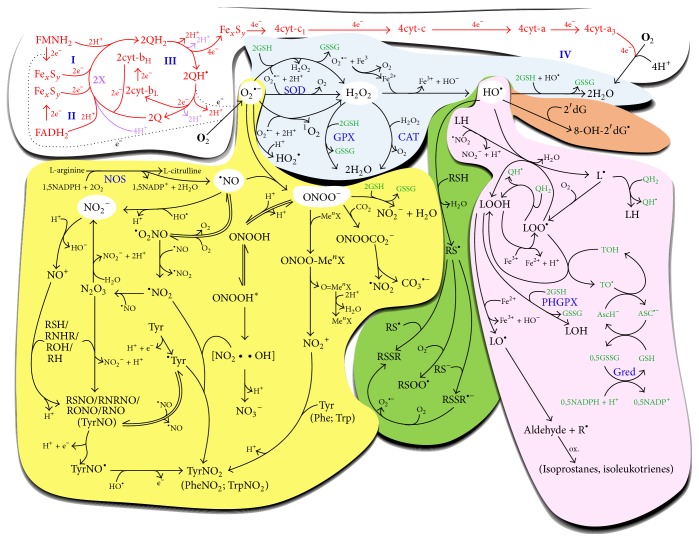
The detailed depiction of the chemistry of mitochondrial oxidative and nitrative/nitrosative stresses. The contents of the colored bubbles correspond to the processes indicated in the bubbles of the respective color in Figure 2. Figure 3 presents the chemical processes of ETC and the terminal oxidation (white bubble; blue Roman numbers represent the site of the respective respiratory complexes) together with those involved in the generation of ROS (light blue bubble) and RNS (yellow bubble). An overview is given on the most representative molecules involved in oxidative injury to nucleic acids (brown bubble), lipids (pink bubble), and molecules with thiol residues (green bubble), as well as in nitrosylation/nitration of organic macromolecules (yellow bubble). For detailed explanation of oxidative/nitrative/nitrosative (black) and antioxidant (green) processes as well as the function of the ETC (red), we refer to the corresponding sections within the text and the Abbreviations section.

Free radicals are molecules possessing unpaired electrons in their outer orbit. This renders them highly reactive towards organic macromolecules such as carbohydrates, nucleic acids, proteins, and lipids, which suffer “injury” during such a reaction. The main routes of free radical production and subsequent toxic insults are represented in a schematic depiction in Figure 2. Under physiological conditions, the efficiency of reducing oxygen during terminal oxidation is approximately 97–99%, while 1–3% undergo incomplete reduction to superoxide (O_2_^•−^), a highly reactive free radical. O_2_^•−^ can be transformed into hydrogen peroxide (H_2_O_2_) both spontaneously and through a reaction catalyzed by mitochondrial manganese superoxide dismutase (Mn-SOD) in the matrix. H_2_O_2_ normally undergoes degradation by glutathione peroxidase (GPX) and catalase (CAT) enzymes, yielding water. In case of an impaired function of the mitochondrial ETC, leakage of excess electrons from complexes I and III leads to a higher amount of O_2_^•−^ and subsequent H_2_O_2_ production, which when exceeding the degradative capacity of the mitochondria can be transformed into the extremely toxic hydroxyl radical (HO^•^), through reaction with transition metals (Fe^2+^ and Cu^2+^; Fenton reaction). HO^•^ in turn can react with nucleic acids and phospholipids, yielding the formation of further toxic radicals and consequent severe functional impairments of the affected macromolecules. Furthermore, HO^•^ can also react with thiol residues, yielding the formation of multiple reactive free radicals. In addition, O_2_^•−^ can also react with nitric oxide (NO^•^) generated from L-arginine by mitochondrial nitric oxide synthase (mtNOS) enzyme [10]; however, other sources of mitochondrial NO^•^ associated with nitrite reductase activity under hypoxic conditions have also been identified [11–13]. The reaction of NO^•^ with O_2_^•−^ yields the highly toxic peroxynitrite anion (ONOO^−^) in a reaction that is three times as fast as the dismutation of O_2_^•−^ by Mn-SOD. ONOO^−^ can evoke injury to proteins via nitration of tyrosine, tryptophan, and phenylalanine residues by nitronium ion (NO_2_^+^) generated through metal-peroxynitrite (ONOO^−^Me^*n*^X) complexes. Nitration of tyrosine can also be achieved by serial reactions of further pathways involving ONOO^−^ and NO^•^ (see Figure 3 for detailed description). Nitrosylation of thiol, secondary amine, alcohol, and alkane residues are performed via their reaction with nitrosonium ion (NO^+^) or indirectly with dinitrogen trioxide (N_2_O_3_), highly reactive radicals also derived from NO^•^. Furthermore, NO^•^ can directly impair the mitochondrial ETC and OXPHOS via competing with oxygen for the oxygen-binding site of complex IV [14], which leads to a further increase in free radical production.

The toxic radicals described above are known as reactive oxygen species (ROS) and reactive nitrogen species (RNS), and their damaging effects on macromolecules are referred to as oxidative and nitrative/nitrosative stress, respectively. Figure 3 presents a detailed overview on the molecular background of toxic processes related to ROS and RNS.

With mitochondrial ETC being the main source of ROS and RNS production, macromolecular components of the mitochondria are extremely exposed to injury due to oxidative/nitrative/nitrosative stress. Of note, proteins that underwent such a damage are highly susceptible to proteolytic cleavage and degradation [15]. The injury to mitochondrial respiratory complex subunits by impaired efficacy of the ETC has two main consequences: (1) it leads to decreased energy production due to impaired OXPHOS, and (2) it decreases the efficacy of terminal oxidation, which results in increased production of ROS/RNS, generating a vicious circle.

The proximity to the main source of free radical production and the relatively high proportion of coding sequences render the mitochondrial genome particularly sensitive to ROS/RNS-mediated injury [16]. Indeed, the mutation rate of mtDNA relative to nDNA is approximately 10 : 1 [17]. Furthermore, as the ability to cope with oxidative/nitrative/nitrosative stress declines with aging [18], the rate of mtDNA mutations further increases in the elderly [19].

Excessive ROS and RNS accumulation can trigger the opening of mitochondrial permeability transition pores (mtPTP), which on the one hand decreases the mitochondrial membrane potential further aggravating the initial OXHPHOS impairment, and, on the other hand, leads to the release of proapoptotic factors (including apoptosis-inducing factor, procaspase-9, and cytochrome *c*) from the intermembrane space to the cytosol. This is in severe cases followed by cellular death that can be either apoptotic or necrotic, depending on the severity of the initial insult and subsequent energy deprivation [20–22]. It should be noted, however, that being among the most ancient signals between mitochondria and their host cells, both ROS and RNS might have essential physiological functions under physiological conditions.

Defensive processes of the mitochondria to counteract excessive free radical production involve low molecular weight antioxidants (LMWAs), an enzymatic redox apparatus to clear ROS/RNS (e.g., SOD, CAT, GPX, and peroxiredoxin), and an nDNA-encoded repair machinery.

Ubiquinol and tocopherols represent the main groups of lipid-associated LMWAs. Since these molecules are transformed to semiquinone radicals upon reduction of toxic free radicals, the immediate restoration of antioxidant capacity is essential. This process depends on the standard redox potential of LMWAs. For example, reduction of the semiquinone form of lipid-associated tocopherol requires ascorbic acid (vitamin C, identified by Albert Szent-Györgyi) and the subsequent reduction of the produced ascorbic acid radical by glutathione. Therefore, at the end of the process, no free radicals are present. However, there is a need for the restoration of the reduced glutathione, which is mediated by the enzyme glutathione reductase (Gred). Reduced glutathione also participates in antioxidant functions associated with the activities of SOD and GPX (including the phospholipid-associated form (PHGPX) as well), and it is responsible for the detoxification of HO^•^ and ONOO^−^. The proper function of this armatory requires the appropriate load of reducing equivalents (NADH + H^+^, NADPH + H^+^). The above mechanisms are depicted in Figure 3.

A number of evidence link PGC-1*α* to the regulation and activation of mitochondrial antioxidant responses. In a comprehensive study of St-Pierre et al. [6], the expression of PGC-1*α* significantly increased after H_2_O_2_ challenge in 10T1/2 cells, which effect was recapitulated by Irrcher et al. on C_2_C_12_ muscle cells [23]. This effect is in correspondence with our recent findings of significantly increased PGC-1*α* expression in the CNS of mice intoxicated with the neurotoxin 3-nitropropionic acid, an irreversible inhibitor or complex II [24]. Furthermore, RNAi against PGC-1*α* reduced the baseline expression of copper/zinc (Cu/Zn)-SOD, Mn-SOD, and GPX in 10T1/2 cells, whereas the expression of Cu/Zn-SOD, Mn-SOD and peroxisomal CAT was found reduced in the heart and brain of PGC-1*α*-deficient mice [6]. Similarly, overexpression of PGC-1*α* in C_2_C_12_ myotubes displayed increased expression of Mn-SOD and GPX in association with a decreased amount of ROS production [25]. Moreover, PGC-1*α*-deficient fibroblasts exhibited blunted response to ROS challenge and an increased sensitivity to oxidative stress, which was in correspondence with an increased sensitivity of PGC-1*α*-deficient mice to intoxication with 1-methyl-4-phenyl-1,2,3,6-tetrahydropyridine (MPTP), an irreversible inhibitor of mitochondrial respiratory complex I, as well as to that with the excitotoxin, kainate [6].

Mitochondrial repair is now widely-acknowledged as an existing phenomenon, comprising a group of processes that aim to repair deleterious alterations in mtDNA, predominantly due to oxidative injuries. These include enzymatic apparatuses for (1) the hydrolysis of oxidized deoxyribonucleotide triphosphates to prevent mismatch errors, (2) different mechanisms of single- and double-strand break repair, (3) multiple mechanisms of base excision repair, and (4) the degradation of unrepairable mtDNA. The latter is a unique mitochondrion-specific mechanism in eukaryotes, which is enabled by the redundance of mtDNA within the organelle [26]. Certain evidence suggest that, similarly to that seen in nDNA repair, poly(ADP-ribose) polymerase-1 (PARP-1) might play central role of the epigenetic regulation of nDNA-encoded proteins involved in mtDNA repair mechanisms [27].

In case the antioxidant defense and repair systems prove insufficient to protect the organelle, severely damaged mitochondria can be sensed and degraded by a process under the regulation of PINK1 and parkin (mitophagy) [28, 29].

## 4. The Central Role of Mitochondrial Dysfunction in Neurodegenerative Diseases

A number of general observations and considerations explain the special susceptibility of the CNS to suffer injuries due to mitochondrial disturbances. Indeed, the CNS has an especially high energy demand as it represents merely 2% of the total body mass and accounts for some 20% of bodily oxygen consumption [30]. Besides, unlike astrocytes, neurons store low amounts of glycogen and have a poor ability to enhance glycolysis under conditions when mitochondrial respiration is impaired [31]. Therefore, neurons depend on the constant availability of oxygen and glucose to maintain their functions. Furthermore, the CNS contains high amounts of polyunsaturated lipids, which are highly susceptible to oxidative injury by means of lipid peroxidation, and the antioxidant capacity of neurons is known to be relatively poor [32, 33]. The high sensitivity of neurons as opposed to the relative resistance of astrocytes to oxygen or glucose deprivation is well known; however, recent studies suggest that oligodendrocytes are among the most sensitive cell types within the CNS to mitochondrial stress, exceeding the vulnerability of neurons [34, 35], a feature that may have implications for the pathogenesis of characteristic myelinopathies in chronic conditions with mitochondrial dysfunction, including aging, and mitochondrial encephalopathies.

Another CNS-specific mechanism leading to an increased sensitivity to mitochondrial dysfunction is excitotoxicity due to glutamate, the major excitatory neurotransmitter in the brain [36]. In an event of energy deprivation, neurons undergo partial membrane depolarization, which removes magnesium ions that block the ionophore of* N*-methyl-D-aspartate-sensitive (NMDA) glutamate receptors. This leads to a persistent activation of NMDA receptors by glutamate even if it is present in physiological levels [37]. Hyperactivation of NMDA receptors results in an influx of calcium into the cytosol. The persistent increase in intracellular calcium level leads to an increased mitochondrial sequestration of calcium, which in pathological extents evokes the opening of high-conductance mtPTPs [38], resulting in mitochondrial swelling and a decreased mitochondrial membrane potential, with subsequent OXPHOS impairment and ROS overproduction [39]. These culminate in the release of proapoptotic factors eventually triggering cell death [40] by apoptotic or necrotic mechanisms, based on the severity of the event [20, 21]. It has also been postulated, however, that the mechanism and the channel (i.e., NMDA receptor) through which calcium ions get into the cell and not the calcium overload itself may play the pivotal roles in excitotoxic cell death [41]. Indeed, NMDA receptors are functionally and spatially linked to neuronal NOS (nNOS) by postsynaptic density protein of molecular weight 95 kDa (PSD-95) that synthesize NO^•^ in a toxic amount while calcium ions enter into the cell during NMDA receptor overactivation [42]. Notably PSD-95 attaches to the NR2B subunit, which is in correspondence with the observation that glutamate excitotoxicity is predominantly mediated by NR2B subunit-containing NMDA receptors [43], which are mainly located extrasynaptically [44]. In line with these, activation of extrasynaptic NMDA receptors is regarded as neurotoxic, whereas that of the synaptic NMDA receptors appears to be neuroprotective [45, 46], indicating a role of volume transmission in NMDA receptor-mediated neurotoxicity. A central role of nNOS in excitotoxic injury was suggested by earlier studies as well [47, 48]. The role of excitotoxicity in association with mitochondrial dysfunction as well as the possible therapeutic relevance of approaches aiming to counteract glutamatergic overactivation in neurodegenerative diseases, including pharmacological manipulations with the kynurenine system, have recently been extensively reviewed [49, 50] and are not within the scope of this review.

The following subsections emphasize the relevance of mitochondrial dysfunction in the pathomechanism of degenerative CNS disorders through a detailed overview on the involvement of impaired OXPHOS and mitochondrial bioenergetics in Parkinson's disease, Huntington's disease, and mitochondrial encephalopathies, with special focus on the involvement and therapeutic relevance of PGC-1*α*.

### 4.1. Parkinson's Disease

**Figure 4:**
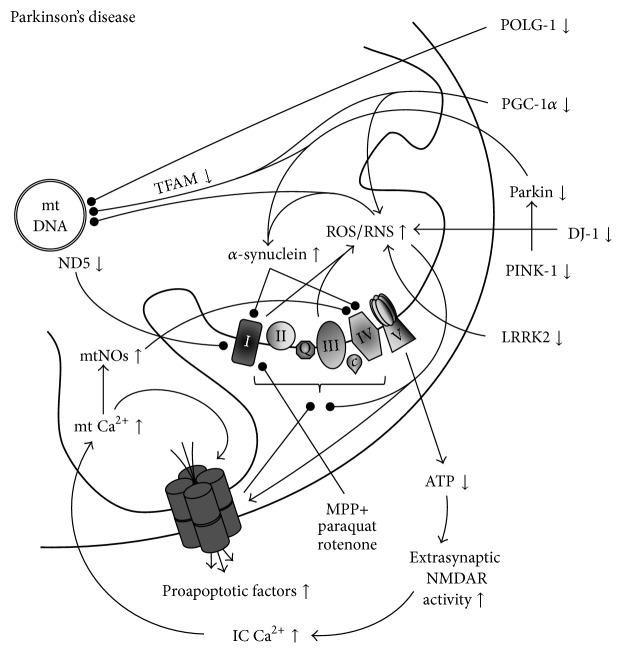
The involvement of mitochondrial dysfunction in Parkinson's disease. Complex I deficiency, the predominant electron transport disorder in sporadic PD has long been linked to the deleterious effects of *α*-synuclein aggregation, a pathognomonic alteration in PD, and inhibitors of complex I (such as MPTP, rotenone, and paraquat) are used in experimental modeling of the disease. Since then a number of genes have been associated with familial forms of the disease, many of them having direct implications in mitochondrial dysfunction. Disturbed OXPHOS in the affected cells can lead to the development of a vicious circle, eventually leading to cell death. Novel findings link PGC-1*α* dysfunction to the pathogenesis of sporadic PD, the restoration of which may hold therapeutic value. (↑ = increased presence/expression/activity; ↓ = decreased presence/expression/activity; arrow = promotion; bulb-headed arrow = inhibition/deterioration.)

Parkinson's disease (PD) is a progressive, chronic neurodegenerative disorder, the pathognomonic alterations of which include loss of dopaminergic neurons, and the presence of Lewy bodies in the substantia nigra pars compacta (SNpc), with a subsequent decrease in striatal dopamine levels [51]. Leading clinical symptoms include bradykinesia, rigidity, resting tremor, and postural instability [52, 53], eventually evolving into severe akinesia, dementia, and eventually death. The development of sporadic PD is linked to a complex interplay of genetic and environmental factors, which have multiple implications for mitochondrial involvement (Figure 4). The first implication for the role of mitochondrial dysfunction in PD came from serial cases of intoxication by the side-product of a synthetic illicit drug, MPTP, which evokes parkinsonian symptoms and recapitulates the majority of PD-related pathologies [54]. Its active metabolite 1-methyl-4-phenylpyridinium (MPP^+^) selectively and irreversibly impairs the function of mitochondrial complex I in dopaminergic neurons [55, 56], and since its discovery, systemic MPTP or intraventricular MPP^+^ intoxication became the most widely applied* in vivo* toxin models of PD. Similar effects can be achieved by known environmental chemicals including the herbicide paraquat and the insecticide rotenone [57]. Corresponding with the ability of complex I inhibitors to evoke parkinsonism, a decreased activity and/or expression of respiratory complex I has been detected in the SNpc [58, 59], striatum [60] frontal cortex [61], platelets [62, 63], and skeletal muscle [64, 65] of sporadic PD patients, suggesting a systemic impairment of mitochondrial functions in this disease. Less consistent reports have been published regarding the involvement of other respiratory complexes, which indicates a predominant involvement of complex I in sporadic PD. In line with these, a decreased activity of complex I, an elevated production of ROS, an energy impairment, and an increased sensitivity to MPP^+^ intoxication can be detected in PD cybrids [66, 67]. The increased presence of oxidative damage has also been reported in post mortem SN of PD patients [68, 69].

In the past decades, genetic and, more recently, genome-wide association studies (GWAS) have identified over 20 loci in causative association with familial PD [70], many of them having direct implications in mitochondrial dysfunction. Among them, leucine-rich repeat kinase 2 (LRRK2) protein is known to colocalize with membrane bound intracellular structures including mitochondria [71]. Its autosomal dominantly inherited mutation, G2019S, the most frequent underlying genetic alteration in both familial and sporadic PD cases, has been associated with mitochondrial dysfunction and morphological alterations in PD tissue samples [72], abnormal mitochondrial dynamics and increased ROS production in murine primary cortical neurons [73], as well as with an increased neuronal vulnerability to rotenone and paraquat in a nematode model of PD [74].

The autosomal dominantly inherited mutation of SNCA gene (A53T) leads to mitochondrial accumulation of *α*-synuclein, the main constituent of Lewy bodies, resulting in the inhibition of respiratory complex I [75, 76]. The protein appears to play pivotal roles in modulating oxidative stress, as its transgenic overexpression leads to enhanced sensitivity against intoxication with paraquat and MPTP [77], whereas *α*-synuclein-deficiency leads to resistance against intoxication with MPTP, 3-nitropropionic acid and malonate in mice [78] (the latter two are inhibitors of complex II and serve as toxin models for Huntington's disease). Accordingly, cell lines transfected with mutant *α*-synuclein exhibit increased levels of oxidation products, decreased glutathione levels, and a markedly increased cell death in response to toxic insults including H_2_O_2_ and MPP^+^ exposure [79].

Among genes associated with an autosomal recessive inheritance of familial PD, parkin, a ubiquitin E3 ligase, is responsible for the polyubiquitin tagging of toxic protein aggregates for proteasomal degradation [80]. In addition, parkin appears to be involved in antioxidant functions through regulating SOD activity and glutathione levels [81] and may play important roles in mitochondrial transcription via its association with mitochondrial transcription factor A (Tfam) [82]. Accordingly, parkin-deficient mice display decreased expression of complex I and IV subunits accompanied by a diminished antioxidant capacity and enhanced oxidative damage [83], whereas parkin-deficient flies develop abnormal mitochondria and exhibit an increased vulnerability to paraquat [84]. Among* in vitro* conditions, overexpression of wild-type parkin reduced, whereas that of the mutant allele aggravated cell death induced by different oxidative stimuli including H_2_O_2_ and MPP^+^ intoxication, which aggravation was accompanied by increased levels of oxidative stress markers and a decreased amount of glutathione [85]. In line with these, transfection of cell lines with mutant parkin leads to an increased presence of markers of oxidative and nitrative injuries [86]. Notably, however, the potential of wild-type parkin overexpression to protect against oxidative insults* in vitro* has been challenged [87], and another study found no increase in vulnerability to different regimens of MPTP exposure in parkin-deficient mice [88].

Parkin appears to share common pathway with phosphatase and tensin homologue- (PTEN-) induced putative kinase 1 (PINK1), another protein associated with autosomal recessive PD, with PINK1 acting upstream of parkin [89]. Indeed, both proteins are involved in the regulation mitochondrial dynamics [28, 29, 90, 91], and phenotypes associated with PINK1-deficiency have been repeatedly reported to be rescued by parkin [89, 92–94]. In experimental models, PINK1-deficiency has been associated with impaired mitochondrial respiration (most consistently complex I deficiency) [95, 96], decreased energy production [92, 93, 97, 98], elevated ROS production [98, 99], impaired mitochondrial calcium handling [98–100], mitochondrial morphological alterations [28, 29, 89–91, 93, 98], and an increased susceptibility to mPTP [99–101]. Furthermore, PINK1-deficiency exacerbates neurodegeneration evoked by MPP^+^ *in vitro* and MPTP* in vivo* [102]. In addition to parkin, tumor necrosis factor (TNF) receptor-associated protein 1 (TRAP1), a mitochondrial molecular chaperone also known as heat shock protein 75 (Hsp75), has been postulated to be another possible downstream target of PINK1, through which PINK1 activity can prevent the release of cytochrome *c* and a subsequent apoptosis [103]. The functional association between TRAP1 and PINK1 has gained further support by more recent studies [104, 105], consistently suggesting that TRAP1 acts downstream of PINK1 and in parallel with parkin when mediating amelioration in mitochondrial dysfunction.

Mutations of DJ-1, an oxidative stress sensor capable of modulating glutathione metabolism and mitochondrial transcription under mitochondrial stress [106], leads to autosomal recessive familial PD. The protein is suggested to function in parallel with PINK1/parkin pathway in maintaining mitochondrial function among oxidative conditions [107]. At the experimental level, DJ-1-deficiency has been associated with increased ROS production [108–110], impaired mitochondrial respiration [109] or energy production [110], mitochondrial morphological abnormalities [109], increased opening of the mPTP [110], as well as an increased sensitivity to oxidative stressors including MPTP [111], paraquat [112], and H_2_O_2_ [112].

Apart from genes identified in monogenic familial PD, a number of genes have been associated with the development of sporadic PD as modifying or susceptibility factors, including genes involved in mitochondrial functions such as mtDNA polymerase gamma 1 (POLG1) [113, 114] and complex I subunit ND5 [115]. In addition to these, an increasing body of evidence suggests that PGC-1*α* may add important contributions to the pathogenesis of PD. Indeed, a comprehensive genome-wide meta-analysis found a set of 425 PGC-1*α*-responsive nuclear-encoded mitochondrial genes underexpressed in sporadic PD, representing pinpoint defects in glucose metabolism and mitochondrial ETC [116]. Furthermore, associations of single nucleotide polymorphisms (SNPs) of PGC-1*α* have been reported with the risk of PD, the age of onset and the longevity [117]. These appear to be in correspondence with decreased expression of PGC-1*α* and its target gene NRF-1 in the SN and striatum of PD patients as well as in the midbrain of conditional parkin knockout mice [118]. In line with decreased ATP production and impairments in mitochondrial OXPHOS [119] and antioxidant responses [6], PGC-1*α*-deficient mice display enhanced susceptibility to MPTP toxicity [6]. Corresponding to observations that mitochondrial dysfunction can promote the aggregation of *α*-synuclein [120], reduced expression of PGC-1*α in vitro* lead to enhanced *α*-synuclein oligomerization [121]. This effect was, however, not confirmed in PGC-1*α*-deficient mice, suggesting a more complex scenario for mitochondrial dysfunction-induced *α*-synuclein aggregation* in vivo* [122]. Supporting a potential therapeutic relevance in PD, overexpression of PGC-1*α* demonstrated neuroprotection against *α*-synuclein- and rotenone-induced toxicity* in vitro* [116] and in a parkin interacting substrate (PARIS) overexpression model of PD* in vivo* [118]. In line with these, transgenic overexpression and resveratrol-induced activation of PGC-1*α* (via deacetylation by Sirt-1) both rendered neuroprotection against MPTP toxicity in mice [123]. Similarly, the administration of pioglitazone, an agonist of PPAR*γ* that enhances the activity and expression of PGC-1*α* [124], was also protective in MPTP studies [125, 126]. These altogether suggest that a deficient expression and/or function of PGC-1*α* and its target genes may play important roles in the development of sporadic PD, which may be of therapeutic relevance in the future. Notably, however, contrasting results have also been published reporting that adenoviral overexpression of PGC-1*α* aggravated MPTP-mediated damage in mice [127] and was ineffective against mutant *α*-synuclein-mediated toxicity in rats [128] and that a sustained overexpression of PGC-1*α* to high levels* per se* evoked the degeneration of nigral neurons in rats [128]. These findings necessitate further investigations and draw the attention to the possibility that a sustained overactivation of mitochondrial biogenesis aiming to restore mitochondrial functions may also have deleterious consequences on the long run. This issue needs to be clarified in the future.

The search for effective neuroprotective compounds capable of modifying the disease course in PD is still extensive; as molecules targeting mitochondrial dysfunction in PD though provided promising results in experimental models of PD [129–131], they were ineffective in clinical trials [132]. In line with the data on a decreased function of PGC-1*α* in PD and on the therapeutic potential of its activation, there is a hope that transcriptional activation of mitochondrial biogenesis and antioxidant responses via PGC-1*α* activation may hold therapeutic value. A phase II safety and futility clinical trial with the PPAR*γ* agonist pioglitazone on patients with early PD has recently been completed, and the results are about to be published in the near future (NCT01280123).

### 4.2. Huntington's Disease

**Figure 5:**
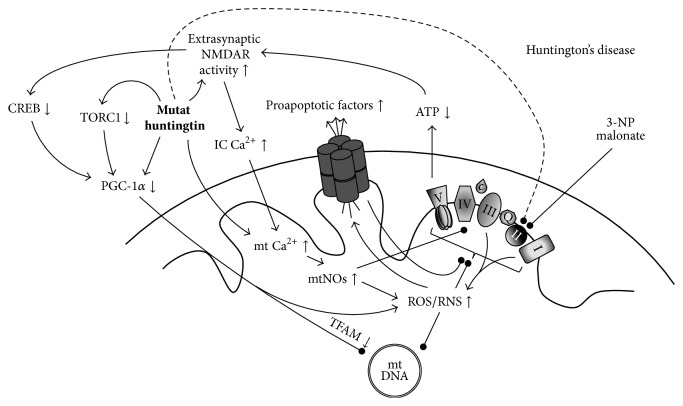
The involvement of mitochondrial dysfunction in Huntington's disease. Complex II deficiency, the predominant electron transport disorder in HD has long been linked to the deleterious effects of mutant huntingtin aggregation, a pathognomonic alteration in HD, and inhibitors of complex II (such as 3-nitropropionic acid (3-NP) and malonate) are used in experimental modeling of the disease. Disturbed OXPHOS in the affected cells can lead to the development of a vicious circle, eventually leading to cell death. Novel findings link PGC-1*α* dysfunction to the pathogenesis of HD at multiple levels, the restoration of which may hold therapeutic value. (↑ = increased presence/expression/activity; ↓ = decreased presence/expression/activity; arrow = promotion; bulb-headed arrow = inhibition/deterioration.)

Huntington's disease (HD) is a monogenic, progressive neurodegenerative disease of autosomal dominant inheritance. The genetic alteration is the expansion of CAG trinucleotide repeat sequence on the interesting transcript 15 (IT15) gene on chromosome 4 encoding huntingtin, with increasing number of repeat associating with earlier onset and more rapid progression [133, 134]. The disease onset is usually between 40 and 50 years of age, presenting with behavioral alterations and hyperkinesia in the early stages, subsequently associating with pyramidal symptoms, dystonia, dementia, and psychosis. The pathognomonic alteration is the preferential loss of the striatal *γ*-aminobutyric acid (GABA)-ergic medium-sized spiny projection neurons (MSNs) and the presence of intracytoplasmic and intranuclear protein inclusions of mutant huntingtin widely distributed in neuronal as well as extraneuronal tissues. The characteristic decreased activity of respiratory complex II, especially in the striatum, has early linked HD to mitochondrial dysfunction [135] (Figure 5). Since then, deficiency in complex II is the most consistent and predominant alteration reported in HD; however, the involvement of other respiratory complexes has also been suggested [136, 137]. The concept of mitochondrial dysfunction mediating the pathological process induced by mutant huntingtin is consistent with an increased presence of oxidative stress [137–141], which is well-reflected by the increased amount of mtDNA mutations observed in HD patients [142]. Further alterations supporting the primary role of mitochondrial dysfunction in mediating the effects of mutant huntingtin include a transcriptional [143] and/or functional [144] repression of PGC-1*α*, disturbances in mitochondrial trafficking [145], a gradually decreasing mitochondrial number [146], and an impairment of mitochondrial calcium handling [147] with an enhanced sensitivity to calcium-induced opening of mPTP and cytochrome* c*-mediated cell death [148, 149].

In line with the predominant biochemical alteration, irreversible inhibition of complex II by 3-nitropropionic acid effectively recapitulates most of the clinical and histopathological characteristics of HD, including the preferential neuronal loss of GABAergic MSNs within the striatum [150, 151]. Similar alterations can be evoked via reversible complex II blockade by malonate [152]. The relevance of complex II dysfunction in HD is highlighted by the fact that mutant huntingtin leads to decreased expression (at the protein level) of the 30 kDa iron-sulfur (Ip) subunit and the 70 kDa FAD (Fp) subunit of complex II in the striatum of HD patients [153] and* in vitro* lentiviral models [153, 154]. Rather similar pattern of alterations was found to underlie complex II deficiency in transgenic HD mice and in a lentiviral model of HD in rats [155]. Complex II dysfunction appears to be causative in HD as its overexpression demonstrated marked restorative effect in these models [153–155]. Alterations in complex II assembly were accompanied by reduced mitochondrial biogenesis in transgenic animals, prompting the authors to suggest the possible contribution of PGC-1*α* repression [155]. Indeed the expression of PGC-1*α* has been found to be downregulated in the striatum of HD patients [143, 144, 146, 156], in transgenic HD animals [24, 143, 144, 157–159], and in* in vitro* HD models [143, 156, 159, 160]. Correspondingly, the decreased expression of several PGC-1*α* target genes has been identified in the striatum of HD patients [144, 146] and transgenic HD mice [144, 157, 158]. A possible mechanism through which mutant huntingtin can lead to the downregulation of PGC-1*α* can be secondary to its effect to enhance the expression [161] and activity of NR2B subunit-containing NMDARs [162], features characteristic of transgenic HD mice [163], which in turn results in a decreased striatal CREB signaling [163], and a subsequent downregulation of PGC-1*α* [164]. In addition, a decreased expression of transducer of regulated CREB-binding protein 1 (TORC1), an activator of CREB-mediated PGC-1*α* expression, has been found in* post mortem* HD striatum, in transgenic HD mice, and in an* in vitro* HD model, which may contribute to the downregulation of PGC-1*α* expression in HD [160], whereas others suggest that PGC-1*α* repression may be secondary to the downregulation of PPAR*γ* in HD [159]. Supplementing the alterations in PGC-1*α* expression in HD, Johri et al. reported decreased protein level of the functionally active N-truncated splice variant of PGC-1*α* (NT-PGC-1*α*) in the striatum of low-grade HD patients and young asymptomatic transgenic N171-82Q and R6/2 HD mice, whereas its level was found to be elevated in high-grade HD patients and in older symptomatic transgenic HD mice [156]. This pattern has been recently supported by our study on transgenic N171-82Q HD mice at the mRNA level, revealing a significantly upregulated expression of NT-PGC-1*α* in both the striatum and overlying cortex of older symptomatic HD mice compared to wild-type and young HD counterparts [24]. These changes were accompanied by a decreased expression of full-length PGC-1*α* in the striatum and cortex of young transgenic mice, corresponding to prior observations. A main novelty of this study included a previously unidentified consistent elevation of both the full-length and the N-truncated isoforms of PGC-1*α* in the cerebellum of transgenic HD mice, which may underlie the relative resistance of cerebellar neurons to degeneration in HD [24]. This study further provided evidence for a consistent striatal upregulation of both the full-length and the N-truncated isoforms of PGC-1*α* following acute but not chronic injury due to 3-nitropropionic acid intoxication in mice [24]. This possibly compensatory elevation corresponds to prior findings of PGC-1*α* upregulation in response to ROS challenge* in vitro* and highlights the role of PGC-1*α* in concerting antioxidant responses [6, 23]. Supporting a potential therapeutic relevance, a line of evidence suggests that mechanisms associated with the upregulation of PGC-1*α* can exert neuroprotection in experimental models of HD. Indeed, the administration of PPAR*γ* agonist thiazolidinediones (such as rosiglitazone and pioglitazone) was proven to be protective in transgenic HD mice [158, 159, 165], in an intrastriatal quinolinic acid-induced rat toxin model of HD [166], in 3-nitropropropionic acid-induced murine toxin model of HD [167], and in* in vitro* HD models [159, 165, 168]. Similarly, the pan-PPAR agonist bezafibrate exerted protection in transgenic HD mice [169]. Furthermore, TORC1 activation displayed protective and restorative effects on viability and mitochondrial functions in a striatal HD cell line model exposed to 3-nitropropionic acid [160]. The protective effect of resveratrol, a polyphenol with potent Sirt-1/PGC-1*α*-activating properties, has also been demonstrated in transgenic murine and nematode models [170], in a 3-nitropropion acid-induced murine model [171], and in an* in vitro* model of HD [170]. Notably, however, the potency of resveratrol to significantly elevate the expression of PGC-1*α* and its target genes within the striatum has recently been questioned [172], which necessitates further investigations. Considering that early attempts with mitochondria-targeted molecules being neuroprotective in experimental HD models [131, 173, 174] provided little success at the clinical level [132], the transcriptional activation of mitochondrial respiration and biogenesis may hold novel therapeutic potential as a more system-based approach. A phase III clinical trial with resveratrol is just about to recruit its participants (NCT02336633).

The corresponding set of evidence implicating a potential pathogenetic role of PGC-1*α* repression in mitochondrial dysfunction in HD as well as early observations of striatal alterations in PGC-1*α*-deficient mice [175, 176] suggested that such animals may serve as experimental models for HD; however, findings of a recent detailed neuropathological evaluation of mice lacking the expression of full-length PGC-1*α* has indicated that it might not indeed be the case and turned our attention to another group of diseases where systemic mitochondrial dysfunction is pathognomonic [122].

### 4.3. Mitochondrial Spongiform Leukoencephalopathies

Mitochondrial diseases are a group of multisystemic disorders where the characteristic pathologies affecting organs with high energy demand (i.e., brain, liver, heart, skeletal muscle, and kidney) are due to mitochondrial dysfunction as a consequence of a genetic alteration either in the mtDNA or in the nDNA. The deleterious loss of functions may affect several components of proper mitochondrial functioning, including genes encoding respiratory complex subunits, proteins responsible for mtDNA transcription/translation, mitochondrial tRNAs and rRNAs, and nuclear-encoded ancillary proteins of mitochondrial function [177]. The diseases are distributed to characteristic syndromes based on the clinical manifestation and the observed neuropathological alterations, including Kearns-Sayre syndrome, Leigh syndrome, mitochondrial encephalomyopathy, lactic acidosis and stroke-like episodes (MELAS), myoclonic epilepsy with ragged-red fibres (MERRF), neuropathy, ataxia, retinitis pigmentosa (NARP), and mitochondrial neurogastrointestinal encephalopathy (MNGIE) [178–181]. In these diseases, impaired ATP production with various defects in respiratory complexes and excess ROS production in the affected tissues has widely been documented and has excessively been reviewed [182]. Though in somewhat different patterns, mitochondrial encephalopathies are collectively characterized by various degrees of tissue vacuolation in the white and gray matter of the CNS, accompanied by region-selective reactive astrocytosis with or without neurodegeneration.

A number of genetically modified murine strains have been developed to model diseases with mitochondrial defects, however, with variable outcomes [122]. On the one hand, many of the genetic modifications lead to embryonic or early postnatal mortality due to multisystemic insufficiency (e.g., complete knockouts of CREB [183], Tfam [184], NRF-1 [185], NRF-2 [186], ERR*γ* [187], POLG1 [188], synthesis of cytochrome *c* oxidase 2 (SCO2) [189], and optic atrophy 1 (OPA1) [190]). On the other hand, a remarkable proportion of viable models, surprisingly, does not have any pathological changes in the CNS (e.g., complete knockouts of adenine nucleotide translocator 1 (ANT1) [191], PPAR*γ* [192], ERR*α* [193], and SURF1 [194]; ΔmtDNA Mito-Mice [195]; and Twinkle mutant “Deletor” mice [196]). Genetic models exhibiting a neuropathology closely reminiscent of human mitochondrial leukoencephalopathies include mice deficient in Mn-SOD [197], in thymidine phosphorylase and uridine phosphorylase (TP/UP) [198], and in NADH dehydrogenase [ubiquinone] iron-sulfur protein 4 (NDUFS4) [199]. In addition to these, our recent neuropathological analysis on mice deficient in the expression of full-length PGC-1*α* revealed widespread spongy vacuolation predominating in the white matter of the striatum, thalamus, cerebellum, and the brainstem, accompanied by moderate to severe reactive astrogliosis in the pontomedullary brainstem and the cerebellar nuclei, corresponding to a pattern of alterations characteristic of the spongiform leukoencephalopathy seen in Kearns-Sayre syndrome [122]. This is especially interesting in light of the facts that experimental animals used for modeling cardiomyopathy in Kearns-Sayre syndrome are the tissue-specific knockouts of Tfam [200, 201], a gene under the regulation PGC-1*α*, and the expression of which is severely downregulated in PGC-1*α*-deficient mice [119, 175]. Notably, no indirect or direct signs indicative striatal neuronal degeneration and/or loss were observed in our study [122], which corresponds to the independent observations of Lucas et al. [202], both publications drawing the conclusion that PGC-1*α*-deficiency* per se* is not sufficient to evoke HD-like pathology, contrasting to what had previously been suggested.

Considering the spectrum of roles of PGC-1*α* in regulating and promoting mitochondrial functions and the fact that a number of genes involved in disease-causing mutations and/or that involved in modeling mitochondrial disease have direct or indirect interactions with PGC-1*α* (e.g., ANT-1, POLG1, Tfam, NRF-1, NRF-2, PPARs, ERRs, Mn-SOD, and CREB), the rationale for PGC-1*α* induction to provide symptomatic benefit in these currently intractable groups of diseases can be accepted [203]. Indeed, transgenic overexpression or bezafibrate-induced expression of PGC-1*α* delayed the onset of symptoms in a cytochrome *c* oxidase-deficient murine model of mitochondrial myopathy [204]. Similarly, transgenic overexpression of PGC-1*α* ameliorated the phenotype and increased the activity of mitochondrial respiratory complexes in POLG1 mutant “Mutator” mice [205]. Furthermore, adenoviral overexpression of PGC-1*α* partially restored respiratory deficits in fibroblasts obtained from patients with mitochondrial disease of various origin (though to different efficacy) and in MELAS cybrids [206]. These altogether suggest a potential therapeutic relevance of boosting mitochondrial biogenesis via PGC-1*α*-mediated approaches in diseases with genetic mitochondrial disorder.

## 5. Concluding Remarks

Since the revelation of the essential role electrons, originating from reducing equivalents that arise from cytosolic and/or mitochondrial metabolic processes, in cellular bioenergetics via their flow through metal-containing electron transport complexes in the mitochondrial inner membrane, an armada of evidence has accumulated linking the impaired function of this system to degenerative diseases of the CNS. While initial attempts to compensate for such alterations showed as much promise at the experimental level as deep disappointment they caused at the clinical level, novel strategies with more system-based approaches aiming to render protection via improving mitochondrial bioenergetics at a transcriptional level may open up new therapeutic perspectives and boost pharmacological research. With more and more evidence linking PGC-1*α* and its target genes to the pathogenesis of neurodegenerative diseases including PD, HD, and mitochondrial disorders, pharmacological manipulations to restore and/or activate PGC-1*α* may provide valuable tools in the therapy of these currently intractable diseases.

## Abbreviations

I: First complex in the mitochondrial ETC
II: Second complex in the mitochondrial ETC
III: Third complex in the mitochondrial ETC
IV: Fourth complex in the mitochondrial ETC
FMNH_2_: Reduced flavin mononucleotide
FADH_2_: Reduced flavin adenine dinucleotide
Fe_*x*_S_*y*_: Iron-sulfur cluster
cyt-a: Cytochrome a
cyt-a_3_: Cytochrome a_3_
cyt-b_H_: Cytochrome bH
cyt-b_L_: Cytochrome bL
cyt-c: Cytochrome c
cyt-c_1_: Cytochrome c_1_
O_2_^•−^: Superoxide anion
Q: Coenzyme Q10 (oxidized)
QH^∙^: Coenzyme Q10 (semiquinone)
QH_2_: Coenzyme Q10 (reduced)
GSH: Glutathione (reduced)
GSSG: Glutathione (oxidized)
SOD: Superoxide dismutase
GPX: Glutathione peroxidase
CAT: Catalase
^1^O_2_: Singlet oxygen
HO^−^: Hydroxide ion
HO^∙^: Hydroxyl radical
HO_2_^∙^: Hydroperoxyl radical
NOS: Nitrogen monoxide synthase
NADPH: Nicotinamide adenine dinucleotide phosphate (reduced)
NADP^+^: Nicotinamide adenine dinucleotide phosphate (oxidized)
NO_2_^−^: Nitrite ion
^∙^NO: Nitrogen monoxide
NO^+^: Nitrosonium ion
^∙^O_2_NO: Nitrite radical
^∙^NO_2_: Nitrogen dioxide radical
N_2_O_3_: Dinitrogen trioxide
ONOO^−^: Peroxynitrite
ONOOH: Peroxynitrous acid
ONOOH^*∗*^: Peroxynitrous acid (metastable)
NO_3_^−^: Nitrate
RSH: Thiol (reduced)
ROH: Alcohol
RNHR: Secondary amine
RH: Alkane
Tyr: Tyrosine
^∙^Tyr: Tyrosyl radical
RSNO: S-Nitrosothiol
RNRNO: Nitrosamine
RONO: Nitrosyl alcohol
RNO: Nitrosoalkane
TyrNO_2_: 3-Nitro-L-tyrosine
PheNO_2_: 3-Nitro-L-phenylalanine
TrpNO_2_: 6-Nitro-L-tryptophan
TyrNO^∙^: 3-Nitrosotyrosine radical
TyrNO: 3-Nitrosotyrosine
Phe: L-Phenylalanine
Trp: L-Tryptophan
NO_2_^+^: Nitronium ion
Me^*n*^X: Metal complexes
O=Me^*n*^X: Oxo-metal complexes
ONOOCO_2_^−^: Nitrosoperoxycarbonate
ONOO-Me^*n*^X: Metal-peroxynitrite complexes
RS^∙^: Thiyl radical
RSSR: Thiol (oxidized)
RSOO^∙^: Thiol peroxyl radical
RSSR^∙−^: Disulphide radical anion
RS^−^: Thiolate anion
LH: Lipid
L^∙^: Lipid radical
LOOH: Lipid peroxide
LOO^∙^: Lipid peroxyl radical
R^∙^: Alkyl radical
LOH: Lipid hydroperoxide
LO^∙^: Lipid alkoxy radical
Gred: Glutathione reductase
PHGPX: Phospholipid glutathione peroxidase
TOH: Tocopherol (reduced)
TO^∙^: Tocopherol (semiquinone)
AscH^−^: Ascorbate (reduced)
Asc^∙−^: Ascorbate (oxidized)
2′dG: 2′-Deoxyguanosine
8-OH-2′dG^∙^: 8-Hydroxy-2′-deoxyguanosine.

## References

[1] Sagan L. (1967). On the origin of mitosing cells. *Journal of Theoretical Biology*.

[2] Sas K., Párdutz Á., Toldi J., Vécsei L. (2010). Dementia, stroke and migraine—some common pathological mechanisms. *Journal of the Neurological Sciences*.

[3] Sas K., Robotka H., Toldi J., Vécsei L. (2007). Mitochondria, metabolic disturbances, oxidative stress and the kynurenine system, with focus on neurodegenerative disorders. *Journal of the Neurological Sciences*.

[4] Mitchell P., Moyle J. (1967). Chemiosmotic hypothesis of oxidative phosphorylation. *Nature*.

[5] Scarpulla R. C. (2008). Transcriptional paradigms in mammalian mitochondrial biogenesis and function. *Physiological Reviews*.

[6] St-Pierre J., Drori S., Uldry M. (2006). Suppression of reactive oxygen species and neurodegeneration by the PGC-1 transcriptional coactivators. *Cell*.

[7] Cantó C., Jiang L. Q., Deshmukh A. S. (2010). Interdependence of AMPK and SIRT1 for metabolic adaptation to fasting and exercise in skeletal muscle. *Cell Metabolism*.

[8] Oliveira N. R., Marques S. O., Luciano T. F. (2014). Treadmill training increases SIRT-1 and PGC-1 alpha protein levels and AMPK phosphorylation in quadriceps of middle-aged rats in an intensity-dependent manner. *Mediators of Inflammation*.

[9] Chen D., Bruno J., Easlon E. (2008). Tissue-specific regulation of SIRT1 by calorie restriction. *Genes & Development*.

[10] Ghafourifar P., Cadenas E. (2005). Mitochondrial nitric oxide synthase. *Trends in Pharmacological Sciences*.

[11] Kozlov A. V., Staniek K., Nohl H. (1999). Nitrite reductase activity is a novel function of mammalian mitochondria. *FEBS Letters*.

[12] Castello P. R., David P. S., McClure T., Crook Z., Poyton R. O. (2006). Mitochondrial cytochrome oxidase produces nitric oxide under hypoxic conditions: implications for oxygen sensing and hypoxic signaling in eukaryotes. *Cell Metabolism*.

[13] Basu S., Azarova N. A., Font M. D. (2008). Nitrite reductase activity of cytochrome *c*. *The Journal of Biological Chemistry*.

[14] Poderoso J. J., Carreras M. C., Lisdero C., Riobó N., Schöpfer F., Boveris A. (1996). Nitric oxide inhibits electron transfer and increases superoxide radical production in rat heart mitochondria and submitochondrial particles. *Archives of Biochemistry and Biophysics*.

[15] Brookes P. S., Yoon Y., Robotham J. L., Anders M. W., Sheu S.-S. (2004). Calcium, ATP, and ROS: a mitochondrial love-hate triangle. *The American Journal of Physiology—Cell Physiology*.

[16] Yakes F. M., van Houten B. (1997). Mitochondrial DNA damage is more extensive and persists longer than nuclear DNA damage in human cells following oxidative stress. *Proceedings of the National Academy of Sciences of the United States of America*.

[17] Wallace D. C. (1994). Mitochondrial DNA sequence variation in human evolution and disease. *Proceedings of the National Academy of Sciences of the United States of America*.

[18] Mecocci P., MacGarvey U., Kaufman A. E. (1993). Oxidative damage to mitochondrial DNA shows marked age-dependent increases in human brain. *Annals of Neurology*.

[19] Ames B. N., Shigenaga M. K., Hagen T. M. (1995). Mitochondrial decay in aging. *Biochimica et Biophysica Acta*.

[20] Bonfoco E., Krainc D., Ankarcrona M., Nicotera P., Lipton S. A. (1995). Apoptosis and necrosis: two distinct events induced, respectively, by mild and intense insults with N-methyl-D-aspartate or nitric oxide/superoxide in cortical cell cultures. *Proceedings of the National Academy of Sciences of the United States of America*.

[21] Ankarcrona M., Dypbukt J. M., Bonfoco E. (1995). Glutamate-induced neuronal death: a succession of necrosis or apoptosis depending on mitochondrial function. *Neuron*.

[22] Lemasters J. J., Qian T., Bradham C. A. (1999). Mitochondrial dysfunction in the pathogenesis of necrotic and apoptotic cell death. *Journal of Bioenergetics and Biomembranes*.

[23] Irrcher I., Ljubicic V., Hood D. A. (2009). Interactions between ROS and AMP kinase activity in the regulation of PGC-1*α* transcription in skeletal muscle cells. *The American Journal of Physiology—Cell Physiology*.

[24] Török R., Kónya J. A., Zádori D. (2015). mRNA expression levels of PGC-1*α* in a transgenic and a toxin model of Huntington's disease. *Cellular and Molecular Neurobiology*.

[25] Kong X., Wang R., Xue Y. (2010). Sirtuin 3, a new target of PGC-1*α*, plays an important role in the suppression of ROS and mitochondrial biogenesis. *PLoS ONE*.

[26] Alexeyev M., Shokolenko I., Wilson G., LeDoux S. (2013). The maintenance of mitochondrial DNA integrity—critical analysis and update. *Cold Spring Harbor Perspectives in Biology*.

[27] Lapucci A., Pittelli M., Rapizzi E., Felici R., Moroni F., Chiarugi A. (2011). Poly(ADP-ribose) polymerase-1 is a nuclear epigenetic regulator of mitochondrial DNA repair and transcription. *Molecular Pharmacology*.

[28] Poole A. C., Thomas R. E., Yu S., Vincow E. S., Pallanck L. (2010). The mitochondrial fusion-promoting factor mitofusin is a substrate of the PINK1/parkin pathway. *PLoS ONE*.

[29] Ziviani E., Tao R. N., Whitworth A. J. (2010). *Drosophila* parkin requires PINK1 for mitochondrial translocation and ubiquitinates mitofusin. *Proceedings of the National Academy of Sciences of the United States of America*.

[30] Papa S. (1996). Mitochondrial oxidative phosphorylation changes in the life span. Molecular aspects and physiopathological implications. *Biochimica et Biophysica Acta*.

[31] Almeida A., Almeida J., Bolaños J. P., Moncada S. (2001). Different responses of astrocytes and neurons to nitric oxide: the role of glycolytically generated ATP in astrocyte protection. *Proceedings of the National Academy of Sciences of the United States of America*.

[32] Beal M. F. (1995). Aging, energy, and oxidative stress in neurodegenerative diseases. *Annals of Neurology*.

[33] Mariani E., Polidori M. C., Cherubini A., Mecocci P. (2005). Oxidative stress in brain aging, neurodegenerative and vascular diseases: an overview. *Journal of Chromatography B: Analytical Technologies in the Biomedical and Life Sciences*.

[34] Goldberg M. P., Choi D. W. (1993). Combined oxygen and glucose deprivation in cortical cell culture: calcium-dependent and calcium-independent mechanisms of neuronal injury. *The Journal of Neuroscience*.

[35] Dewar D., Underhill S. M., Goldberg M. P. (2003). Oligodendrocytes and ischemic brain injury. *Journal of Cerebral Blood Flow and Metabolism*.

[36] Lau A., Tymianski M. (2010). Glutamate receptors, neurotoxicity and neurodegeneration. *Pflugers Archiv*.

[37] Novelli A., Reilly J. A., Lysko P. G., Henneberry R. C. (1988). Glutamate becomes neurotoxic via the *N*-methyl-D-aspartate receptor when intracellular energy levels are reduced. *Brain Research*.

[38] Ichas F., Mazat J.-P. (1998). From calcium signaling to cell death: two conformations for the mitochondrial permeability transition pore. Switching from low- to high-conductance state. *Biochimica et Biophysica Acta*.

[39] Crompton M. (1999). The mitochondrial permeability transition pore and its role in cell death. *The Biochemical Journal*.

[40] Li P., Nijhawan D., Budihardjo I. (1997). Cytochrome c and dATP-dependent formation of Apaf-1/caspase-9 complex initiates an apoptotic protease cascade. *Cell*.

[41] Tymianski M., Charlton M. P., Carlen P. L., Tator C. H. (1993). Source specificity of early calcium neurotoxicity in cultured embryonic spinal neurons. *The Journal of Neuroscience*.

[42] Sattler R., Xiong Z., Lu W.-Y., Hafner M., MacDonald J. F., Tymianski M. (1999). Specific coupling of NMDA receptor activation to nitric oxide neurotoxicity by PSD-95 protein. *Science*.

[43] Liu Y., Wong T. P., Aarts M. (2007). NMDA receptor subunits have differential roles in mediating excitotoxic neuronal death both in vitro and in vivo. *The Journal of Neuroscience*.

[44] Tovar K. R., Westbrook G. L. (1999). The incorporation of NMDA receptors with a distinct subunit composition at nascent hippocampal synapses in vitro. *The Journal of Neuroscience*.

[45] Hardingham G. E., Fukunaga Y., Bading H. (2002). Extrasynaptic NMDARs oppose synaptic NMDARs by triggering CREB shut-off and cell death pathways. *Nature Neuroscience*.

[46] Léveillé F., El Gaamouch F., Gouix E. (2008). Neuronal viability is controlled by a functional relation between synaptic and extrasynaptic NMDA receptors. *The FASEB Journal*.

[47] Dawson V. L., Dawson T. M., London E. D., Bredt D. S., Snyder S. H. (1991). Nitric oxide mediates glutamate neurotoxicity in primary cortical cultures. *Proceedings of the National Academy of Sciences of the United States of America*.

[48] Dawson V. L., Kizushi V. M., Huang P. L., Snyder S. H., Dawson T. M. (1996). Resistance to neurotoxicity in cortical cultures from neuronal nitric oxide synthase-deficient mice. *The Journal of Neuroscience*.

[49] Zádori D., Klivényi P., Szalárdy L., Fülöp F., Toldi J., Vécsei L. (2012). Mitochondrial disturbances, excitotoxicity, neuroinflammation and kynurenines: novel therapeutic strategies for neurodegenerative disorders. *Journal of the Neurological Sciences*.

[50] Vécsei L., Szalárdy L., Fülöp F., Toldi J. (2013). Kynurenines in the CNS: recent advances and new questions. *Nature Reviews Drug Discovery*.

[51] Forno L. S. (1996). Neuropathology of Parkinson's disease. *Journal of Neuropathology and Experimental Neurology*.

[52] Lang A. E., Lozano A. M. (1998). Parkinson's disease. *The New England Journal of Medicine*.

[53] Lang A. E., Lozano A. M. (1998). Parkinson's disease: second of two parts. *The New England Journal of Medicine*.

[54] Forno L. S., DeLanney L. E., Irwin I., Langston J. W. (1993). Similarities and differences between MPTP-induced parkinsonsim and Parkinson's disease. Neuropathologic considerations. *Advances in Neurology*.

[55] Ramsay R. R., Salach J. I., Dadgar J., Singer T. P. (1986). Inhibition of mitochondrial NADH dehydrogenase by pyridine derivatives and its possible relation to experimental and idiopathic parkinsonism. *Biochemical and Biophysical Research Communications*.

[56] Javitch J. A., D'Amato R. J., Strittmatter S. M., Snyder S. H. (1985). Parkinsonism-inducing neurotoxin, N-methyl-4-phenyl-1,2,3,6-tetrahydropyridine: uptake of the metabolite N-methyl-4-phenylpyridine by dopamine neurons explains selective toxicity. *Proceedings of the National Academy of Sciences of the United States of America*.

[57] Banerjee R., Starkov A. A., Beal M. F., Thomas B. (2009). Mitochondrial dysfunction in the limelight of Parkinson's disease pathogenesis. *Biochimica et Biophysica Acta—Molecular Basis of Disease*.

[58] Schapira A. H. V., Cooper J. M., Dexter D., Clark J. B., Jenner P., Marsden C. D. (1990). Mitochondrial complex I deficiency in Parkinson's disease. *Journal of Neurochemistry*.

[59] Hattori N., Tanaka M., Ozawa T., Mizuno Y. (1991). Immunohistochemical studies on complexes I, II, III, and IV of mitochondria in Parkinson's disease. *Annals of Neurology*.

[60] Mizuno Y., Ohta S., Tanaka M. (1989). Deficiencies in complex I subunits of the respiratory chain in Parkinson's disease. *Biochemical and Biophysical Research Communications*.

[61] Parker W. D., Parks J. K., Swerdlow R. H. (2008). Complex I deficiency in Parkinson's disease frontal cortex. *Brain Research*.

[62] Haas R. H., Nasirian F., Nakano K. (1995). Low platelet mitochondrial complex I and complex II/III activity in early untreated Parkinson's disease. *Annals of Neurology*.

[63] Benecke R., Strumper P., Weiss H. (1993). Electron transfer complexes I and IV of platelets are abnormal in Parkinson's disease but normal in Parkinson-plus syndromes. *Brain*.

[64] Blin O., Desnuelle C., Rascol O. (1994). Mitochondrial respiratory failure in skeletal muscle from patients with Parkinson's disease and multiple system atrophy. *Journal of the Neurological Sciences*.

[65] Bindoff L. A., Birch-Machin M. A., Cartlidge N. E. F., Parker W. D., Turnbull D. M. (1991). Respiratory chain abnormalities in skeletal muscle from patients with Parkinson's disease. *Journal of the Neurological Sciences*.

[66] Swerdlow R. H., Parks J. K., Miller S. W. (1996). Origin and functional consequences of the complex I defect in Parkinson's disease. *Annals of Neurology*.

[67] Esteves A. R. F., Domingues A. F., Ferreira I. L. (2008). Mitochondrial function in Parkinson's disease cybrids containing an nt2 neuron-like nuclear background. *Mitochondrion*.

[68] Alam Z. I., Jenner A., Daniel S. E. (1997). Oxidative DNA damage in the Parkinsonian brain: an apparent selective increase in 8-hydroxyguanine levels in substantia nigra. *Journal of Neurochemistry*.

[69] Zhang J., Perry G., Smith M. A. (1999). Parkinson's disease is associated with oxidative damage to cytoplasmic DNA and RNA in substantia nigra neurons. *The American Journal of Pathology*.

[70] Bonifati V. (2014). Genetics of Parkinson's disease—state of the art, 2013. *Parkinsonism & Related Disorders*.

[71] Biskup S., Moore D. J., Celsi F. (2006). Localization of LRRK2 to membranous and vesicular structures in mammalian brain. *Annals of Neurology*.

[72] Mortiboys H., Johansen K. K., Aasly J. O., Bandmann O. (2010). Mitochondrial impairment in patients with Parkinson disease with the G2019S mutation in LRRK2. *Neurology*.

[73] Niu J., Yu M., Wang C., Xu Z. (2012). Leucine-rich repeat kinase 2 disturbs mitochondrial dynamics via Dynamin-like protein. *Journal of Neurochemistry*.

[74] Saha S., Guillily M. D., Ferree A. (2009). LRRK2 modulates vulnerability to mitochondrial dysfunction in Caenorhabditis elegans. *The Journal of Neuroscience*.

[75] Devi L., Raghavendran V., Prabhu B. M., Avadhani N. G., Anandatheerthavarada H. K. (2008). Mitochondrial import and accumulation of *α*-synuclein impair complex I in human dopaminergic neuronal cultures and Parkinson disease brain. *The Journal of Biological Chemistry*.

[76] Chinta S. J., Mallajosyula J. K., Rane A., Andersen J. K. (2010). Mitochondrial alpha-synuclein accumulation impairs complex I function in dopaminergic neurons and results in increased mitophagy in vivo. *Neuroscience Letters*.

[77] Norris E. H., Uryu K., Leight S., Giasson B. I., Trojanowski J. Q., Lee V. M.-Y. (2007). Pesticide exposure exacerbates *α*-synucleinopathy in an A53T transgenic mouse model. *The American Journal of Pathology*.

[78] Klivenyi P., Siwek D., Gardian G. (2006). Mice lacking alpha-synuclein are resistant to mitochondrial toxins. *Neurobiology of Disease*.

[79] Lee M., Hyun D.-H., Halliwell B., Jenner P. (2001). Effect of the overexpression of wild-type or mutant *α*-synuclein on cell susceptibility to insult. *Journal of Neurochemistry*.

[80] Shimura H., Hattori N., Kubo S.-I. (2000). Familial Parkinson disease gene product, parkin, is a ubiquitin-protein ligase. *Nature Genetics*.

[81] Yang H., Zhou H.-Y., Li B., Niu G.-Z., Chen S.-D. (2007). Downregulation of parkin damages antioxidant defenses and enhances proteasome inhibition-induced toxicity in PC12 cells. *Journal of Neuroimmune Pharmacology*.

[82] Kuroda Y., Mitsui T., Kunishige M. (2006). Parkin enhances mitochondrial biogenesis in proliferating cells. *Human Molecular Genetics*.

[83] Palacino J. J., Sagi D., Goldberg M. S. (2004). Mitochondrial dysfunction and oxidative damage in parkin-deficient mice. *The Journal of Biological Chemistry*.

[84] Pesah Y., Pham T., Burgess H. (2004). *Drosophila* parkin mutants have decreased mass and cell size and increased sensitivity to oxygen radical stress. *Development*.

[85] Hyun D.-H., Lee M., Halliwell B., Jenner P. (2005). Effect of overexpression of wild-type or mutant parkin on the cellular response induced by toxic insults. *Journal of Neuroscience Research*.

[86] Hyun D.-H., Lee M., Hattori N. (2002). Effect of wild-type or mutant parkin on oxidative damage, nitric oxide, antioxidant defenses, and the proteasome. *The Journal of Biological Chemistry*.

[87] Darios F., Corti O., Lücking C. B. (2003). Parkin prevents mitochondrial swelling and cytochrome c release in mitochondria-dependent cell death. *Human Molecular Genetics*.

[88] Thomas B., von Coelln R., Mandir A. S. (2007). MPTP and DSP-4 susceptibility of substantia nigra and locus coeruleus catecholaminergic neurons in mice is independent of parkin activity. *Neurobiology of Disease*.

[89] Clark I. E., Dodson M. W., Jiang C. (2006). *Drosophila* pink1 is required for mitochondrial function and interacts genetically with parkin. *Nature*.

[90] Poole A. C., Thomas R. E., Andrews L. A., McBride H. M., Whitworth A. J., Pallanck L. J. (2008). The PINK1/Parkin pathway regulates mitochondrial morphology. *Proceedings of the National Academy of Sciences of the United States of America*.

[91] Deng H., Dodson M. W., Huang H., Guo M. (2008). The Parkinson's disease genes pink1 and parkin promote mitochondrial fission and/or inhibit fusion in *Drosophila*. *Proceedings of the National Academy of Sciences of the United States of America*.

[92] Exner N., Treske B., Paquet D. (2007). Loss-of-function of human PINK1 results in mitochondrial pathology and can be rescued by parkin. *The Journal of Neuroscience*.

[93] Yang Y., Gehrke S., Imai Y. (2006). Mitochondrial pathology and muscle and dopaminergic neuron degeneration caused by inactivation of *Drosophila* Pink1 is rescued by Parkin. *Proceedings of the National Academy of Sciences of the United States of America*.

[94] Park J., Lee S. B., Lee S. (2006). Mitochondrial dysfunction in *Drosophila PINK1* mutants is complemented by *parkin*. *Nature*.

[95] Gautier C. A., Kitada T., Shen J. (2008). Loss of PINK1 causes mitochondrial functional defects and increased sensitivity to oxidative stress. *Proceedings of the National Academy of Sciences of the United States of America*.

[96] Vilain S., Esposito G., Haddad D. (2012). The yeast complex I equivalent NADH dehydrogenase rescues pink1 mutants. *PLoS Genetics*.

[97] Amo T., Sato S., Saiki S. (2011). Mitochondrial membrane potential decrease caused by loss of PINK1 is not due to proton leak, but to respiratory chain defects. *Neurobiology of Disease*.

[98] Heeman B., van den Haute C., Aelvoet S.-A. (2011). Depletion of PINK1 affects mitochondrial metabolism, calcium homeostasis and energy maintenance. *Journal of Cell Science*.

[99] Gandhi S., Wood-Kaczmar A., Yao Z. (2009). PINK1-associated Parkinson's disease is caused by neuronal vulnerability to calcium-induced cell death. *Molecular Cell*.

[100] Akundi R. S., Huang Z., Eason J. (2011). Increased mitochondrial calcium sensitivity and abnormal expression of innate immunity genes precede dopaminergic defects in Pink1-deficient mice. *PLoS ONE*.

[101] Gautier C. A., Giaime E., Caballero E. (2012). Regulation of mitochondrial permeability transition pore by PINK1. *Molecular Neurodegeneration*.

[102] Haque M. E., Thomas K. J., D'Souza C. (2008). Cytoplasmic Pink1 activity protects neurons from dopaminergic neurotoxin MPTP. *Proceedings of the National Academy of Sciences of the United States of America*.

[103] Pridgeon J. W., Olzmann J. A., Chin L.-S., Li L. (2007). PINK1 protects against oxidative stress by phosphorylating mitochondrial chaperone TRAP1. *PLoS Biology*.

[104] Zhang L., Karsten P., Hamm S. (2013). TRAP1 rescues PINK1 loss-of-function phenotypes. *Human Molecular Genetics*.

[105] Costa A. C., Loh S. H., Martins L. M. (2013). *Drosophila* Trap1 protects against mitochondrial dysfunction in a PINK1/parkin model of Parkinson's disease. *Cell Death & Disease*.

[106] McCoy M. K., Cookson M. R. (2011). DJ-1 regulation of mitochondrial function and autophagy through oxidative stress. *Autophagy*.

[107] Thomas K. J., McCoy M. K., Blackinton J. (2011). DJ-1 acts in parallel to the PINK1/parkin pathway to control mitochondrial function and autophagy. *Human Molecular Genetics*.

[108] Andres-Mateos E., Perier C., Zhang L. (2007). DJ-1 gene deletion reveals that DJ-1 is an atypical peroxiredoxin-like peroxidase. *Proceedings of the National Academy of Sciences of the United States of America*.

[109] Krebiehl G., Ruckerbauer S., Burbulla L. F. (2010). Reduced basal autophagy and impaired mitochondrial dynamics due to loss of Parkinson's disease-associated protein DJ-1. *PLoS ONE*.

[110] Giaime E., Yamaguchi H., Gautier C. A., Kitada T., Shen J. (2012). Loss of DJ-1 does not affect mitochondrial respiration but increases ROS production and mitochondrial permeability transition pore opening. *PLoS ONE*.

[111] Kim R. H., Smith P. D., Aleyasin H. (2005). Hypersensitivity of DJ-1-deficient mice to 1-methyl-4-phenyl-1,2,3,6-tetrahydropyrindine (MPTP) and oxidative stress. *Proceedings of the National Academy of Sciences of the United States of America*.

[112] Meulener M., Whitworth A. J., Armstrong-Gold C. E. (2005). *Drosophila* DJ-1 mutants are selectively sensitive to environmental toxins associated with Parkinson's disease. *Current Biology*.

[113] Davidzon G., Greene P., Mancuso M. (2006). Early-onset familial parkinsonism due to POLG mutations. *Annals of Neurology*.

[114] Luoma P. T., Eerola J., Ahola S. (2007). Mitochondrial DNA polymerase gamma variants in idiopathic sporadic Parkinson disease. *Neurology*.

[115] Parker W. D., Parks J. K. (2005). Mitochondrial ND5 mutations in idiopathic Parkinson's disease. *Biochemical and Biophysical Research Communications*.

[116] Zheng B., Liao Z., Locascio J. J. (2010). PGC-1alpha, a potential therapeutic target for early intervention in Parkinson's disease. *Science Translational Medicine*.

[117] Clark J., Reddy S., Zheng K., Betensky R. A., Simon D. K. (2011). Association of PGC-1alpha polymorphisms with age of onset and risk of Parkinson's disease. *BMC Medical Genetics*.

[118] Shin J.-H., Ko H. S., Kang H. (2011). PARIS (ZNF746) repression of PGC-1*α* contributes to neurodegeneration in Parkinson's disease. *Cell*.

[119] Arany Z., He H., Lin J. (2005). Transcriptional coactivator PGC-1*α* controls the energy state and contractile function of cardiac muscle. *Cell Metabolism*.

[120] Betarbet R., Sherer T. B., MacKenzie G., Garcia-Osuna M., Panov A. V., Greenamyre J. T. (2000). Chronic systemic pesticide exposure reproduces features of Parkinson's disease. *Nature Neuroscience*.

[121] Ebrahim A. S., Ko L.-W., Yen S.-H. (2010). Reduced expression of peroxisome-proliferator activated receptor gamma coactivator-1alpha enhances alpha-synuclein oligomerization and down regulates AKT/GSK3beta signaling pathway in human neuronal cells that inducibly express alpha-synuclein. *Neuroscience Letters*.

[122] Szalardy L., Zadori D., Plangar I. (2013). Neuropathology of partial PGC-1alpha deficiency recapitulates features of mitochondrial encephalopathies but not of neurodegenerative diseases. *Neurodegenerative Diseases*.

[123] Mudò G., Mäkelä J., Di Liberto V. (2012). Transgenic expression and activation of PGC-1*α* protect dopaminergic neurons in the MPTP mouse model of Parkinsons disease. *Cellular and Molecular Life Sciences*.

[124] Hondares E., Mora O., Yubero P. (2006). Thiazolidinediones and rexinoids induce peroxisome proliferator-activated receptor-coactivator (PGC)-1alpha gene transcription: an autoregulatory loop controls PGC-1alpha expression in adipocytes via peroxisome proliferator-activated receptor-gamma coactivation. *Endocrinology*.

[125] Breidert T., Callebert J., Heneka M. T., Landreth G., Launay J. M., Hirsch E. C. (2002). Protective action of the peroxisome proliferator-activated receptor-*γ* agonist pioglitazone in a mouse model of Parkinson's disease. *Journal of Neurochemistry*.

[126] Dehmer T., Heneka M. T., Sastre M., Dichgans J., Schulz J. B. (2004). Protection by pioglitazone in the MPTP model of Parkinson's disease correlates with I kappa B alpha induction and block of NF kappa B and iNOS activation. *Journal of Neurochemistry*.

[127] Clark J., Silvaggi J. M., Kiselak T. (2012). Pgc-1*α* overexpression downregulates Pitx3 and increases susceptibility to MPTP toxicity associated with decreased Bdnf. *PLoS ONE*.

[128] Ciron C., Lengacher S., Dusonchet J., Aebischer P., Schneider B. L. (2012). Sustained expression of PGC-1*α* in the rat nigrostriatal system selectively impairs dopaminergic function. *Human Molecular Genetics*.

[129] Matthews R. T., Ferrante R. J., Klivenyi P. (1999). Creatine and cyclocreatine attenuate MPTP neurotoxicity. *Experimental Neurology*.

[130] Schulz J. B., Henshaw D. R., Matthews R. T., Beal M. F. (1995). Coenzyme Q10 and nicotinamide and a free radical spin trap protect against MPTP neurotoxicity. *Experimental Neurology*.

[131] Yang L., Calingasan N. Y., Wille E. J. (2009). Combination therapy with Coenzyme Q10 and creatine produces additive neuroprotective effects in models of Parkinson's and Huntington's Diseases. *Journal of Neurochemistry*.

[132] Szalárdy L., Klivényi P., Zádori D., Fülöp F., Toldi J., Vécsei L. (2012). Mitochondrial disturbances, tryptophan metabolites and neurodegeneration: medicinal chemistry aspects. *Current Medicinal Chemistry*.

[133] Walker F. O. (2007). Huntington's disease. *The Lancet*.

[134] Hassel B., Tessler S., Faull R. L. M., Emson P. C. (2008). Glutamate uptake is reduced in prefrontal cortex in Huntington's disease. *Neurochemical Research*.

[135] Stahl W. L., Swanson P. D. (1974). Biochemical abnormalities in Huntington's chorea brains. *Neurology*.

[136] Gu M., Gash M. T., Mann V. M., Javoy-Agid F., Cooper J. M., Schapira A. H. V. (1996). Mitochondrial defect in Huntington's disease caudate nucleus. *Annals of Neurology*.

[137] Browne S. E., Bowling A. C., MacGarvey U. (1997). Oxidative damage and metabolic dysfunction in huntington's disease: selective vulnerability of the basal ganglia. *Annals of Neurology*.

[138] Polidori M. C., Mecocci P., Browne S. E., Senin U., Beal M. F. (1999). Oxidative damage to mitochondrial DNA in Huntington's disease parietal cortex. *Neuroscience Letters*.

[139] Chen C.-M., Wu Y.-R., Cheng M.-L. (2007). Increased oxidative damage and mitochondrial abnormalities in the peripheral blood of Huntington's disease patients. *Biochemical and Biophysical Research Communications*.

[140] Long J. D., Matson W. R., Juhl A. R., Leavitt B. R., Paulsen J. S. (2012). 8OHdG as a marker for Huntington disease progression. *Neurobiology of Disease*.

[141] Browne S. E., Ferrante R. J., Beal M. F. (1999). Oxidative stress in Huntington's disease. *Brain Pathology*.

[142] Horton T. M., Graham B. H., Corral-Debrinski M. (1995). Marked increase in mitochondrial DNA deletion levels in the cerebral cortex of Huntington's disease patients. *Neurology*.

[143] Cui L., Jeong H., Borovecki F., Parkhurst C. N., Tanese N., Krainc D. (2006). Transcriptional repression of PGC-1alpha by mutant huntingtin leads to mitochondrial dysfunction and neurodegeneration. *Cell*.

[144] Weydt P., Pineda V. V., Torrence A. E. (2006). Thermoregulatory and metabolic defects in Huntington's disease transgenic mice implicate PGC-1*α* in Huntington's disease neurodegeneration. *Cell Metabolism*.

[145] Chang D. T. W., Rintoul G. L., Pandipati S., Reynolds I. J. (2006). Mutant huntingtin aggregates impair mitochondrial movement and trafficking in cortical neurons. *Neurobiology of Disease*.

[146] Kim J., Moody J. P., Edgerly C. K. (2010). Mitochondrial loss, dysfunction and altered dynamics in Huntington's disease. *Human Molecular Genetics*.

[147] Panov A. V., Gutekunst C.-A., Leavitt B. R. (2002). Early mitochondrial calcium defects in Huntington's disease are a direct effect of polyglutamines. *Nature Neuroscience*.

[148] Sawa A., Wiegand G. W., Cooper J. (1999). Increased apoptosis of Huntington disease lymphoblasts associated with repeat length-dependent mitochondrial depolarization. *Nature Medicine*.

[149] Choo Y. S., Johnson G. V. W., MacDonald M., Detloff P. J., Lesort M. (2004). Mutant huntingtin directly increases susceptibility of mitochondria to the calcium-induced permeability transition and cytochrome c release. *Human Molecular Genetics*.

[150] Gould D. H., Gustine D. L. (1982). Basal ganglia degeneration, myelin alterations, and enzyme inhibition induced in mice by the plant toxin 3-nitropropanoic acid. *Neuropathology and Applied Neurobiology*.

[151] Brouillet E., Condé F., Beal M. F., Hantraye P. (1999). Replicating Huntington's disease phenotype in experimental animals. *Progress in Neurobiology*.

[152] Beal M. F., Brouillet E., Jenkins B., Henshaw R., Rosen B., Hyman B. T. (1993). Age-dependent striatal excitotoxic lesions produced by the endogenous mitochondrial inhibitor malonate. *Journal of Neurochemistry*.

[153] Benchoua A., Trioulier Y., Zala D. (2006). Involvement of mitochondrial complex II defects in neuronal death produced by N-terminus fragment of mutated huntingtin. *Molecular Biology of the Cell*.

[154] Benchoua A., Trioulier Y., Diguet E. (2008). Dopamine determines the vulnerability of striatal neurons to the N-terminal fragment of mutant huntingtin through the regulation of mitochondrial complex II. *Human Molecular Genetics*.

[155] Damiano M., Diguet E., Malgorn C. (2013). A role of mitochondrial complex II defects in genetic models of Huntington's disease expressing N-terminal fragments of mutant huntingtin. *Human Molecular Genetics*.

[156] Johri A., Starkov A. A., Chandra A. (2011). Truncated peroxisome proliferator-activated receptor-gamma coactivator 1alpha splice variant is severely altered in Huntington's disease. *Neurodegenerative Diseases*.

[157] Chaturvedi R. K., Calingasan N. Y., Yang L., Hennessey T., Johri A., Beal M. F. (2010). Impairment of PGC-1alpha expression, neuropathology and hepatic steatosis in a transgenic mouse model of Huntington's disease following chronic energy deprivation. *Human Molecular Genetics*.

[158] Jin J., Albertz J., Guo Z. (2013). Neuroprotective effects of PPAR-*γ* agonist rosiglitazone in N171-82Q mouse model of Huntington's disease. *Journal of Neurochemistry*.

[159] Chiang M. C., Chen C. M., Lee M. R. (2010). Modulation of energy deficiency in Huntington's disease via activation of the peroxisome proliferator-activated receptor gamma. *Human Molecular Genetics*.

[160] Chaturvedi R. K., Hennessey T., Johri A. (2012). Transducer of regulated creb-binding proteins (TORCs) transcription and function is impaired in Huntington's disease. *Human Molecular Genetics*.

[161] Chen N., Luo T., Wellington C. (1999). Subtype-specific enhancement of NMDA receptor currents by mutant huntingtin. *Journal of Neurochemistry*.

[162] Song C., Zhang Y., Parsons C. G., Liu Y. F. (2003). Expression of polyglutamine-expanded huntingtin induces tyrosine phosphorylation of N-methyl-D-aspartate receptors. *The Journal of Biological Chemistry*.

[163] Milnerwood A. J., Gladding C. M., Pouladi M. A. (2010). Early increase in extrasynaptic NMDA receptor signaling and expression contributes to phenotype onset in Huntington's disease mice. *Neuron*.

[164] Okamoto S.-I., Pouladi M. A., Talantova M. (2009). Balance between synaptic versus extrasynaptic NMDA receptor activity influences inclusions and neurotoxicity of mutant huntingtin. *Nature Medicine*.

[165] Chiang M.-C., Chern Y., Huang R.-N. (2012). PPARgamma rescue of the mitochondrial dysfunction in Huntington's disease. *Neurobiology of Disease*.

[166] Kalonia H., Kumar P., Kumar A. (2010). Pioglitazone ameliorates behavioral, biochemical and cellular alterations in quinolinic acid induced neurotoxicity: possible role of peroxisome proliferator activated receptor-*ϒ* (PPAR*ϒ*) in Huntington's disease. *Pharmacology Biochemistry and Behavior*.

[167] Napolitano M., Costa L., Palermo R., Giovenco A., Vacca A., Gulino A. (2011). Protective effect of pioglitazone, a PPAR*γ* ligand, in a 3 nitropropionic acid model of Huntington's disease. *Brain Research Bulletin*.

[168] Quintanilla R. A., Jin Y. N., Fuenzalida K., Bronfman M., Johnson G. V. W. (2008). Rosiglitazone treatment prevents mitochondrial dysfunction in mutant huntingtin-expressing cells: possible role of peroxisome proliferator-activated receptor-gamma (PPARgamma) in the pathogenesis of Huntington disease. *The Journal of Biological Chemistry*.

[169] Johri A., Calingasan N. Y., Hennessey T. M. (2012). Pharmacologic activation of mitochondrial biogenesis exerts widespread beneficial effects in a transgenic mouse model of Huntington's disease. *Human Molecular Genetics*.

[170] Parker J. A., Arango M., Abderrahmane S. (2005). Resveratrol rescues mutant polyglutamine cytotoxicity in nematode and mammalian neurons. *Nature Genetics*.

[171] Kumar P., Padi S. S. V., Naidu P. S., Kumar A. (2006). Effect of resveratrol on 3-nitropropionic acid-induced biochemical and behavioural changes: possible neuroprotective mechanisms. *Behavioural Pharmacology*.

[172] Ho D. J., Calingasan N. Y., Wille E., Dumont M., Beal M. F. (2010). Resveratrol protects against peripheral deficits in a mouse model of Huntington's disease. *Experimental Neurology*.

[173] Vamos E., Voros K., Vecsei L., Klivenyi P. (2010). Neuroprotective effects of L-carnitine in a transgenic animal model of Huntington's disease. *Biomedicine & Pharmacotherapy*.

[174] Matthews R. T., Yang L., Jenkins B. G. (1998). Neuroprotective effects of creatine and cyclocreatine in animal models of Huntington's disease. *The Journal of Neuroscience*.

[175] Leone T. C., Lehman J. J., Finck B. N. (2005). PGC-1alpha deficiency causes multi-system energy metabolic derangements: muscle dysfunction, abnormal weight control and hepatic steatosis. *PLoS Biology*.

[176] Lin J., Wu P.-H., Tarr P. T. (2004). Defects in adaptive energy metabolism with CNS-linked hyperactivity in PGC-1*α* null mice. *Cell*.

[177] Finsterer J., Harbo H. F., Baets J. (2009). EFNS guidelines on the molecular diagnosis of mitochondrial disorders. *European Journal of Neurology*.

[178] Brown G. K., Squier M. V. (1996). Neuropathology and pathogenesis of mitochondrial diseases. *Journal of Inherited Metabolic Disease*.

[179] Tanji K., Kunimatsu T., Vu T. H., Bonilla E. (2001). Neuropathological features of mitochondrial disorders. *Seminars in Cell and Developmental Biology*.

[180] Betts J., Lightowlers R. N., Turnbull D. M. (2004). Neuropathological aspects of mitochondrial DNA disease. *Neurochemical Research*.

[181] Filosto M., Tomelleri G., Tonin P. (2007). Neuropathology of mitochondrial diseases. *Bioscience Reports*.

[182] Federico A., Cardaioli E., Da Pozzo P., Formichi P., Gallus G. N., Radi E. (2012). Mitochondria, oxidative stress and neurodegeneration. *Journal of the Neurological Sciences*.

[183] Rudolph D., Tafuri A., Gass P., Hämmerling G. J., Arnold B., Schütz G. (1998). Impaired fetal T cell development and perinatal lethality in mice lacking the cAMP response element binding protein. *Proceedings of the National Academy of Sciences of the United States of America*.

[184] Larsson N.-G., Wang J., Wilhelmsson H. (1998). Mitochondrial transcription factor A is necessary for mtDNA maintenance and embryogenesis in mice. *Nature Genetics*.

[185] Huo L., Scarpulla R. C. (2001). Mitochondrial DNA instability and peri-implantation lethality associated with targeted disruption of nuclear respiratory factor 1 in mice. *Molecular and Cellular Biology*.

[186] Ristevski S., O'Leary D. A., Thornell A. P., Owen M. J., Kola I., Hertzog P. J. (2004). The ETS transcription factor GABP*α* is essential for early embryogenesis. *Molecular and Cellular Biology*.

[187] Alaynick W. A., Kondo R. P., Xie W. (2007). ERR*γ* directs and maintains the transition to oxidative metabolism in the postnatal heart. *Cell Metabolism*.

[188] Hance N., Ekstrand M. I., Trifunovic A. (2005). Mitochondrial DNA polymerase gamma is essential for mammalian embryogenesis. *Human Molecular Genetics*.

[189] Yang H., Brosel S., Acin-Perez R. (2009). Analysis of mouse models of cytochrome c oxidase deficiency owing to mutations in Sco2. *Human Molecular Genetics*.

[190] Davies V. J., Hollins A. J., Piechota M. J. (2007). Opa1 deficiency in a mouse model of autosomal dominant optic atrophy impairs mitochondrial morphology, optic nerve structure and visual function. *Human Molecular Genetics*.

[191] Lee J., Schriner S. E., Wallace D. C. (2009). Adenine nucleotide translocator 1 deficiency increases resistance of mouse brain and neurons to excitotoxic insults. *Biochimica et Biophysica Acta*.

[192] Zhao X., Strong R., Zhang J. (2009). Neuronal PPARgamma deficiency increases susceptibility to brain damage after cerebral ischemia. *The Journal of Neuroscience*.

[193] Luo J., Sladek R., Carrier J., Bader J.-A., Richard D., Giguère V. (2003). Reduced fat mass in mice lacking orphan nuclear receptor estrogen-related receptor alpha. *Molecular and Cellular Biology*.

[194] Dell'Agnello C., Leo S., Agostino A. (2007). Increased longevity and refractoriness to Ca^2+^-dependent neurodegeneration in Surf1 knockout mice. *Human Molecular Genetics*.

[195] Tanaka D., Nakada K., Takao K. (2008). Normal mitochondrial respiratory function is essential for spatial remote memory in mice. *Molecular Brain*.

[196] Tyynismaa H., Mjosund K. P., Wanrooij S. (2005). Mutant mitochondrial helicase Twinkle causes multiple mtDNA deletions and a late-onset mitochondrial disease in mice. *Proceedings of the National Academy of Sciences of the United States of America*.

[197] Melov S., Schneider J. A., Day B. J. (1998). A novel neurological phenotype in mice lacking mitochondrial manganese superoxide dismutase. *Nature Genetics*.

[198] López L. C., Akman H. O., García-Cazorla Á. (2009). Unbalanced deoxynucleotide pools cause mitochondrial DNA instability in thymidine phosphorylase-deficient mice. *Human Molecular Genetics*.

[199] Quintana A., Kruse S. E., Kapur R. P., Sanz E., Palmiter R. D. (2010). Complex I deficiency due to loss of Ndufs4 in the brain results in progressive encephalopathy resembling Leigh syndrome. *Proceedings of the National Academy of Sciences of the United States of America*.

[200] Li H., Wang J., Wilhelmsson H. (2000). Genetic modification of survival in tissue-specific knockout mice with mitochondrial cardiomyopathy. *Proceedings of the National Academy of Sciences of the United States of America*.

[201] Wang J., Wilhelmsson H., Graff C. (1999). Dilated cardiomyopathy and atrioventricular conduction blocks induced by heart-specific inactivation of mitochondrial DNA gene expression. *Nature Genetics*.

[202] Lucas E. K., Dougherty S. E., McMeekin L. J., Trinh A. T., Reid C. S., Cowell R. M. (2012). Developmental alterations in motor coordination and medium spiny neuron markers in mice lacking PGC-1alpha. *PLoS ONE*.

[203] Wenz T. (2009). PGC-1*α* activation as a therapeutic approach in mitochondrial disease. *IUBMB Life*.

[204] Wenz T., Diaz F., Spiegelman B. M., Moraes C. T. (2008). Activation of the PPAR/PGC-1*α* pathway prevents a bioenergetic deficit and effectively improves a mitochondrial myopathy phenotype. *Cell Metabolism*.

[205] Dillon L. M., Williams S. L., Hida A. (2012). Increased mitochondrial biogenesis in muscle improves aging phenotypes in the mtDNA mutator mouse. *Human Molecular Genetics*.

[206] Srivastava S., Diaz F., Iommarini L., Aure K., Lombes A., Moraes C. T. (2009). PGC-1alpha/beta induced expression partially compensates for respiratory chain defects in cells from patients with mitochondrial disorders. *Human Molecular Genetics*.

